# The role of synovial fluid aspiration in shoulder joint infections

**DOI:** 10.1186/s12891-022-05285-x

**Published:** 2022-04-26

**Authors:** Laura Elisa Streck, Johannes Forster, Sebastian Philipp von Hertzberg-Boelch, Thomas Reichel, Maximilian Rudert, Kilian Rueckl

**Affiliations:** 1 Department of Orthopedic Surgery, Koenig-Ludwig-Haus, University of Wuerzburg, Brettreichstrasse 11, 97074 Wuerzburg, Germany; 2 Insititute for Hygiene and Microbiology, University of Wuerzburg, Josef-Schneider-Strasse 2, 97080 Wuerzburg, Germany

**Keywords:** Revision arthroplasty, Periprosthetic joint infection, white blood cell count, Septic, Microbiological culture, Interstage aspiration

## Abstract

**Background:**

Joint aspiration with analysis of synovial fluid white blood cell count (WBC) and microbiological culture is a widely established aspect in the diagnosis of shoulder joint infections (SJI). In case of a two stage revision for SJI, joint aspiration before re−/implantation of a total shoulder arthroplasty (TSA) was used to rule out persistent infection for years but its value is under debate. Shoulder specific data on all aspects is rare. The current study aims to answer the following research questions: Does joint aspiration have an insufficient predictive value in the diagnosis of SJI in (1) initial workup and (2) before definite arthroplasty with polymethylmethacrylate (PMMA)-Spacer in place?

**Methods:**

This retrospective evaluation investigates 35 patients that were treated for SJI with a two staged implantation of a TSA after debridement and implantation of an PMMA-Spacer. Joint aspirations were performed preoperatively (PA) and before re−/implantation of the prosthesis while spacer was in place (interstage aspiration, IA). Samples were taken for microbiological culture and analysis of WBC. Sensitivity and specificity were calculated with reference to intraoperative microbiological samples. Receiver Operating Characteristic (ROC), Area-Under-Curve analysis (AUC) and calculation of the Youden index were performed to find optimum cut-off for WBC.

**Results:**

The sensitivity of microbiological cultures from PA was 58.3% and the specificity was 88.9%. The mean WBC was 27,800 leucocytes/mm^3^ (range 400-96,300). The maximum Youden index (0.857) was a cut-off of 2600 leucocytes/mm^3^ with a sensitivity of 85.7% and a specificity of 100.0%. The sensitivity and specificity of IA were 0.0% and 88.5%, respectively.

**Conclusions:**

Preoperative aspiration is likely to miss Cutibacteria *spp*. and CoNS and cannot rule out infection for sure. However, we recommend it for its advantages of targeted antibiotic therapy in case of germ identification. Empiric antibiotic therapy should cover Cutibacteria and CoNS even if aspiration showed negative microbiological cultures. In contrast, the diagnostic value of interstage aspiration does not qualify for its routine use.

## Background

Joint infections are a serious condition both after surgical intervention as well as in the native joint. They often result in multiple revision surgeries, relevant loss of function in the affected extremity and are accompanied by a high mortality [1–3]. The early and reliable diagnosis of an infection is essential for effective treatment and prevention of generalized, septic progressions [1–6]. Synovial fluid aspiration is a central pillar in the diagnostic workup. Diagnostic markers from synovial fluid include white blood cell count (WBC) with polymorphonuclear percentage, leukocyte esterase level, alpha-defensin level, synovial CRP and microbiological culture. WBC has been used as a reliable tool in the diagnosis of infections of the lower extremity but shoulder specific data is limited to scattered and small studies [7]. To date, there is no consensus about cut-off values. The direct transfer of values from the lower extremity does not seem to be adequate [8]. The specific bacterial spectrum with Cutibacterium *spp*. and coagulase-negative staphylococci (CoNS) further complicates the assessment of results of microbiological cultures and increases the risk of false negative results [3, 9, 10]. The reported sensitivity of PA widely varies between 9 and 85% and its value is under debate [9–13].

This uncertainty applies even more for joint aspiration with PMMA-spacer in place during two-stage revision surgery, a common therapy regimen for periprosthetic shoulder infections [14–16]. It was common practice to perform a joint aspiration after 14 days of antibiotic suspension and await the microbiologic culture before final TSA implantation. This procedure has widely been abandoned in two-stage revision for periprosthetic infections of the hip and knee as sensitivity and specificity of this “interstage aspiration” (IA) were reported low [17–20]. Shoulder specific data is largely absent. However, it is doubtful whether a transfer of the experience gained in hip and knee surgery to shoulder infections is justified. Therefore, it is crucial to gather joint specific data on SJI to improve diagnostic tools, scores and workflows.

The current study aims to answer the following research questions: Does joint aspiration have an insufficient predictive value in the diagnosis of shoulder joint infections (SJI) in (1) initial workup and (2) before definite arthroplasty with PMMA-Spacer in place?

## Materials and methods

This retrospective evaluation investigated consecutive patients that were treated for SJI with a two staged implantation of a total shoulder arthroplasty (TSA) after debridement and implantation of an antibiotic loaded PMMA-Spacer between 2007 and 2015 in one specialized high volume hospital. Inclusion criteria were 1) the diagnosis of SJI based on clinical presentation, blood infection markers, histological and microbiological findings as well as WBC following 2018s ICM criteria [21], 2) the two staged TSA implantation as described above and 3) available data on PA and IA. Exclusion criteria was age below 18. We identified 35 patients that were eligible for the study (23 periprosthetic shoulder infections (PSI), 8 native joint infections (primary infection, PI), 7 infections after osteosynthesis and/or rotator cuff surgery (secondary infection, SI). The first joint aspiration was performed preoperatively (PA); the second aspiration was performed as interstage aspiration while a spacer was implanted (IA). Patients did not receive antibiotics before PA. All patients were treated with targeted (if available) or empiric intravenous antibiotics for 14 days after spacer implantation followed by oral administration. An antibiotic suspension of 14 days was held before performing IA. The implanted antibiotic loaded PMMA-Spacer was handmade around a bent Steinmann-Pin using Palacos R + G (Heraeus Medical, Wehrheim, Germany) with manually added Vancomycin in 34 cases and Copal G + C (Heraeus Medical, Wehrheim, Germany) in 1 case.

Data for certain aspects was not available in all of the patients, therefore sample sizes differ for different questions. Culture from PA: 35 patients (7 PI, 21 PSI, 8 SI), WBC: 12 patients (3 PI, 9 PSI), IA: 33 patients (6 PI, 21 PSI, 7 SI). Details on demographic data and blood infection markers are provided in Table 1.

**Table 1:** Demographic data and preoperative blood infection markers

Demographic data and blood infection markers									
Patient	Sex category	Site	Initial surgery	Age at PA [years]	Surgery to PA [months]	Spacer to IA [days]	CRP [mg/dl]	ESR [mm/h]	Blood WBC [10^**3**^/mm^**3**^]
1	female	right	osteosynthesis	44	18	55	1.6	34	7.6
2	female	right	no surgery	74		48	4.7	67	6.1
3	female	left	no surgery	71		32	2.5		10.5
4	malefte	right	TSA	55	35	34	8.5	23	9.5
5	female	right	TSA	71	14	15	4		9.5
6	female	right	TSA	80	32	40	6.8	82	8.8
7	female	left	osteosynthesis	68	4	52	2.9	64	10.7
8	female	right	TSA	57	11	58	2.4	31	12.2
9	male	left	osteosynthesis	52	8	52	1.5		7.1
10	female	right	TSA	84	4	57		21	
11	male	right	TSA	59	155	38	1.2		7.8
12	male	right	rotator cuff	68	32	44	1.3	18	8.6
13	female	right	TSA	77	56	318^a^	0.4		6.6
14	female	right	osteosynthesis	66	2	50	1.1		5.4
15	female	left	TSA	74	16	46	0.4		9.4
16	male	left	TSA	74	13	58	1.7	18	11.4
17	male	right	TSA	55	10	41	2.6	64	8.1
18	male	right	TSA	73	24	44	11.3	32	8.3
19	male	right	TSA	84	8	86	1.5	40	4.4
20	female	right	TSA	65	14	50	4.8		8.8
21	male	right	TSA	72	1	74	30.4	68	8.6
22	male	right	TSA	76	2	48	10	44	11.1
23	female	right	TSA	60	9	44	1.4		6.6
24	female	left	TSA	78	37	56	12.1	40	13.8
25	female	right	no surgery	76		332^a^	1.6		7.2
26	female	right	TSA	77	28	56	0.1	38	5.6
27	female	right	no surgery	82		41	1.9	29	7.6
28	female	right	no surgery	84		20	7.1	8	10.3
29	female	right	TSA	69	101	58	0.1	24	7.5
30	female	left	no surgery	83		59	0.3		8.3
31	female	right	no surgery	79			6.6	64	6
32	female	left	osteosynthesis	65	2	59	1	31	10.5
33	female	left	TSA	84	69	56	1.8	6	11.7
34	male	left	rotator cuff	50	127		0	29	5.4
35	female	right	TSA	80	10	58	9.7	1	21.8
Mean (only patients with TSA)				72	31	47^b^	5.5	35	9.5
Mean (patients with non TSA)				69	28	51^b^	2.4	38	7.9
Mean (total)				70	30	49^b^	4.3	37	8.9

*CRP* C-reactive protein (norm value < 0,8 mg/dl), *ESR* Erythrocyte sedimentation rate (norm value < 28 mm/h)

^a^IA was delayed due to intermediate cardic surgery/treatment for pulmonary problems. These two patients were excluded for the calculation of means (marked with ^b^)

Joint aspirations were performed with a sterile canula from either anterior or dorsal approach after 3 minutes of skin disinfection by alcoholic skin disinfectant (Octeniderm®, Schuelke and May, Norderstedt, Germany). A minimum volume of 1 ml synovial fluid was required. Aspirated fluid volume was never diluted by using other fluids. Aspirated synovial fluid was transferred to a sterile test tube for WBC calculation and to blood culture bottles for aerobic (BacT/ALERT® FA Plus) and anaerobic (BacT/ALERT® FN Plus) growth (bioMérieux, Marvie-L’Étoile, France). Blood culture vials were incubated for 14 days or until flagged positive in the BacT/ALERT® 3D System (bioMérieux, Marvie-L’Étoile, France). Species diagnosis was established by Matrix-assisted laser desorption/ionization – time of flight (MALDI-TOF) from solid culture media.

Statistical tests were performed using SPSS Statistics® Version 24.0 (IBM, New York, USA) and Microsoft Excel® Version 1908 (Microsoft, Washington, USA). Descriptive statistics were performed to describe means, medians and range for all variables. Receiver Operating Characteristic (ROC) was plotted for WBC depending on culture results from spacer implantation to depict the correlation between sensitivity and specificity. Area-Under-Curve (AUC) analysis and calculation of the Youden index were performed to quantify the quality of the test. The same calculations were performed for WBC depending on culture results from PA.

Microbiological culture from PA was defined as “correct positive” if a microbiological culture from tissue biopsies taken during initial surgery matched the organism of PA. PA was defined “correct negative” if neither the culture from PA nor intraoperative biopsies yielded growth. PA was defined “false negative” if the culture from PA was negative while an intraoperative biopsy yielded growth.

PA was defined “false positive” if culture from PA yielded growth and intraoperative biopsies yielded growth of a different bacterium (1 patient) or no growth.

The intraoperative biopsies were taken right after opening the joint capsule and before starting perioperative intravenous antibiotics. At least 3 biopsies were taken from membranes, resected bone and directly surrounding soft tissue. Sterile sample vessels were opened under laminar flow and fractioned. Cultures were nourished in Brain-Heart-Infusion-Bouillon and Thyoglycolate-Bouillon and plated out on Candida-Chrome-Agar, Columbia-Blood-Agar, Cooking-Blood-Agar, MacConkey-Agar and Schaedler-Agar. Cultures were incubated for 14 days. Species were differentiated throughout mass spectrometry (Vitek MS, bioMérieux, Marvie-L’Étoile, France) followed by testing of sensitivity (Vitek 2, bioMérieux, Marvie-L’Étoile, France).

Sensitivity was defined as the ratio of all correct positive PAs on all intraoperative positive cultures. Specificity was defined as the ratio of all positive PAs on all intraoperative positive cultures. The same procedure was used for IAs but with reference to results from either spacer exchange or final implantation of TSA. Cross-tables were used to calculate positive predictive value (PPV) and negative predictive value (NPV) for both PA and IA.

## Results

### Microbiological culture from preoperative aspiration

**Fig. 1:**
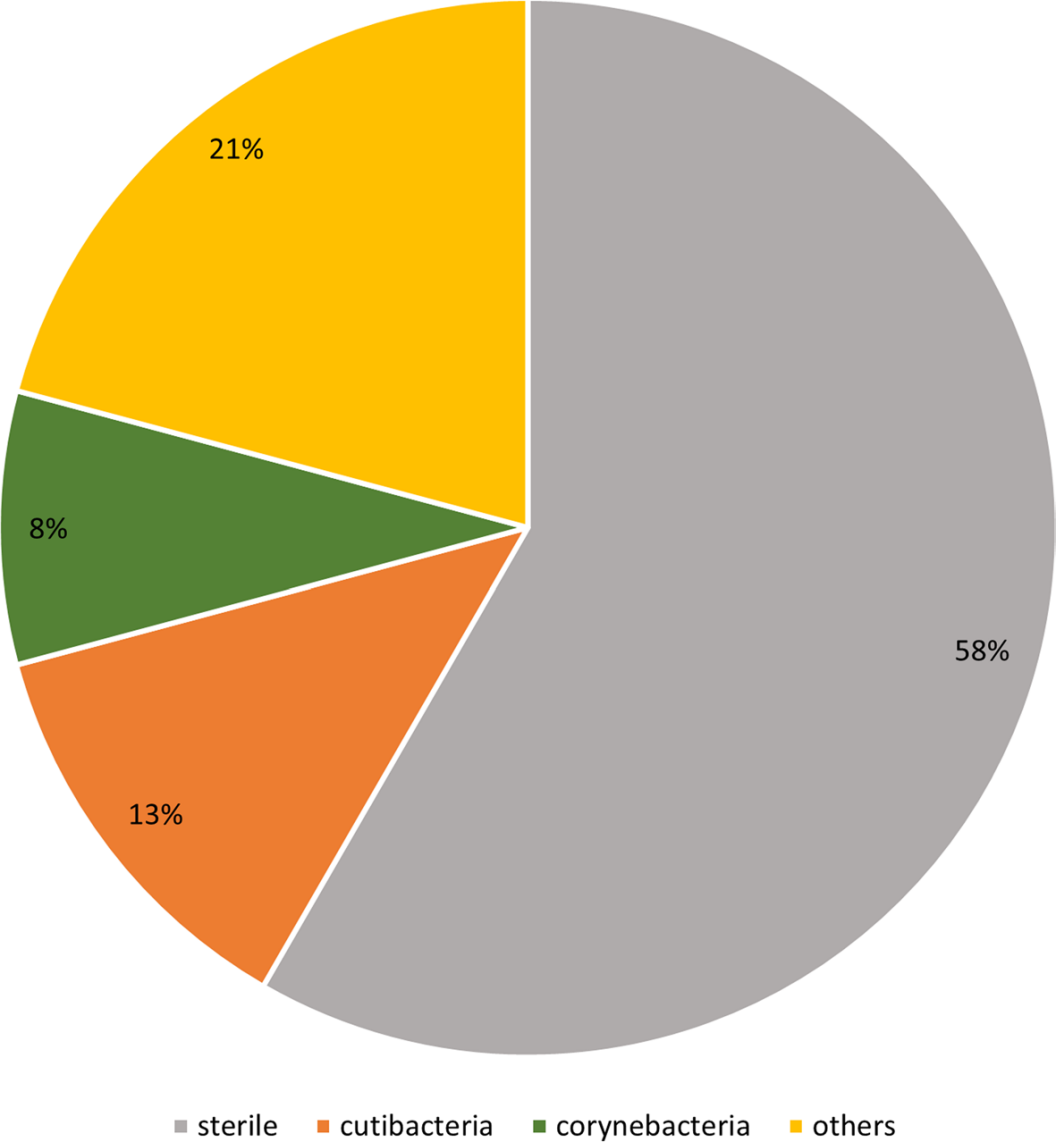
Microbiological cultures from PA Results of microbiological cultures from PA (in % of all samples). For detailed information on detected bacteria see text

PA did not provide required minimum fluid volume in 11/35 cases. The remaining 24 cases had positive cultures in 42% of the cases. Within this positive cultures, bacteria detected was 30% Cutibacterium *spp.,* 20% Corynebacterium *spp.* and 50% others (Enterococcus *faecalis*, Escherichia *coli*, Staphylococcus* aureus*, Streptococcus* viridans* in 10% respectively and further 10% bacteria who yielded obligate anaerobe growth in blood culture bottles but species diagnose was impeded by insufficient growth on solid media). Figure 1 depicts results of microbiological cultures from PA. Sensitivity was 58.3%, specificity was 88.9%. PPV was 0.88, NPV was 0.64. Table 2 depicts detailed information on microbiological results.

**Table 2:** WBC and results of microbiological cultures in patients with shoulder joint infections

Synovial WBC and microbiological culture results						
Patient	Initial surgery	WBC from PA [cells/mm^**3**^]	Culture from PA	Culture from spacer implantation	Culture from IA	Culture from spacer exchange/TSA implantation
1	osteosynthesis		insufficient	CoNS	negative	
2	no surgery		insufficient	S.* epidermidis*	negative	negative
3	no surgery		negative		negative	negative
4	TSA	400	negative	Cutibacteria	negative	negative
5	TSA		insufficient	S.* epidermidis*	negative	negative
6	TSA		E.* faecalis*		insufficient	negative
7	osteosynthesis		Corynebacteria *spp.*		negative	negative
8	TSA		anaerobic bacteria	F.* magna*	Paracoccus *spp.*	negative
9	osteosynthesis		insufficient	S.* epidermidis*	negative	negative
10	TSA		negative	S.* aureus*	negative	negative
11	TSA		insufficient	negative	negative	
12	rotator cuff		negative	negative	negative	negative
13	TSA		insufficient	Cutibacteria	negative	negative
14	osteosynthesis		insufficient	S.* epidermidis* + C. *acnes*	negative	negative
15	TSA	400	E.* coli*	negative	negative	negative
16	TSA	23,400	negative	S.* epidermidis*	negative	negative
17	TSA		negative	negative	negative	C.* acnes*
18	TSA		negative	C. *acnes*	Paenibacillus* spp.*	negative
19	TSA		insufficient	C. *acnes*	negative	S.* warneri*
20	TSA	56,200	Corynebacteria *spp.*	C.* avidum*	S.* epidermidis*	negative
21	TSA		insufficient	gramnegative strains	negative	negative
22	TSA	76,800	C.* acnes*	C.* acnes*	negative	negative
23	TSA	36,600	S.* aureus*	S.* aureus*	negative	negative
24	TSA	96,300	S.* viridans*	S.* viridans*	negative	negative
25	no surgery		negative	negative	negative	negative
26	TSA		negative	S.* xylosus*	negative	negative
27	no surgery		insufficient	negative	negative	negative
28	no surgery	1200	negative	negative	negative	negative
29	TSA	700	negative	negative	insufficient	S.* epidermidis*
30	no surgery	2600	negative	negative	negative	negative
31	no surgery	400	negative	negative		negative
32	osteosynthesis		insufficient	S.* epidermidis*	negative	negative
33	TSA	45,000	C.* avidum*	C.* avidum*	negative	negative
34	rotator cuff		C.* acnes*	C.* acnes*		negative
35	TSA		negative	negative	insufficient	S.* epidermidis*

### Synovial fluid white blood cell count

**Fig. 2:**
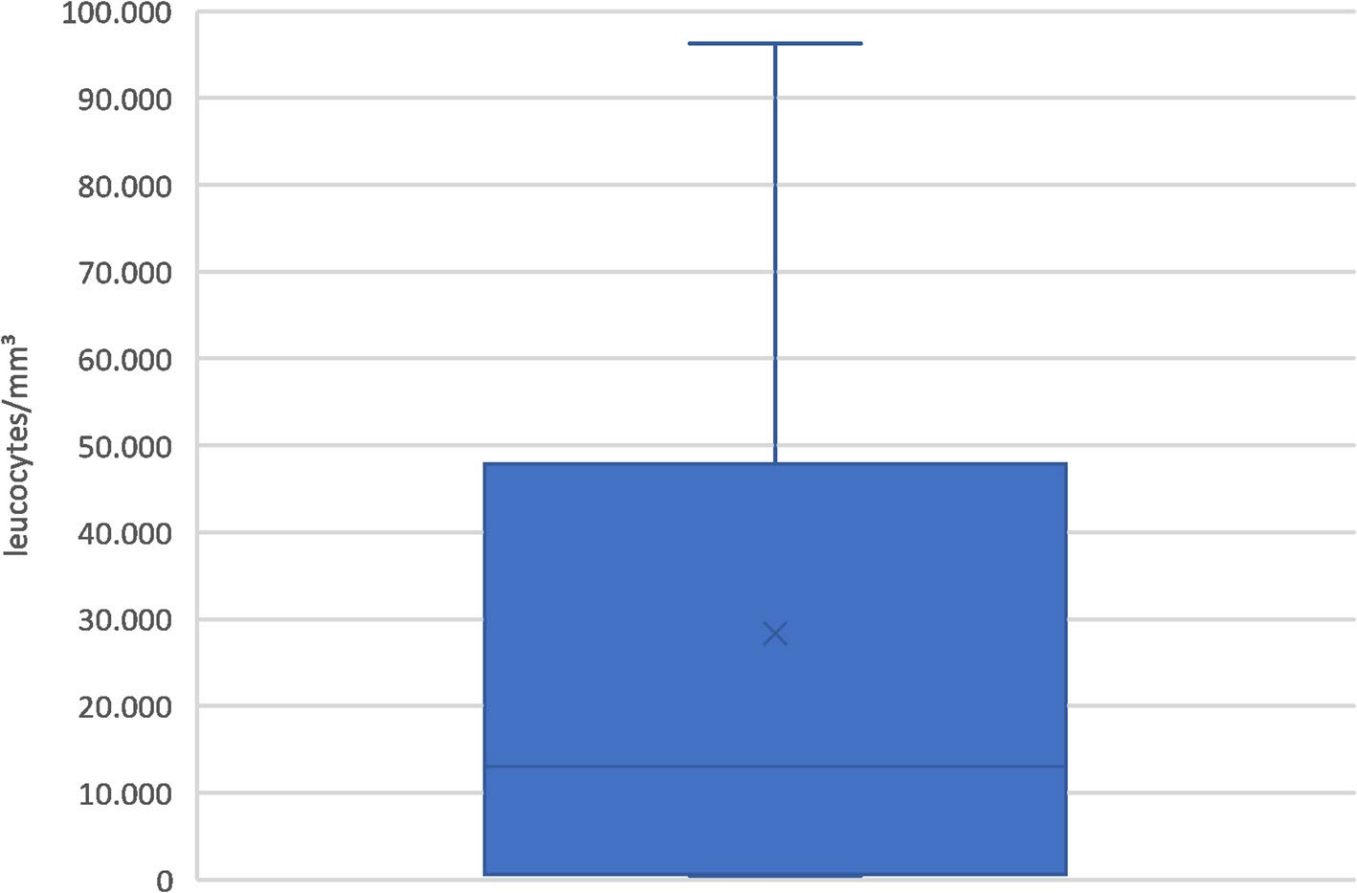
WBC. Synovial fluid white blood cell count in preoperative joint aspiration (WBC) in leucocytes/mm^3^. Mean WBC was 27,800 leucocytes/mm^3^ (range 400-96,300)

**Fig. 3:**
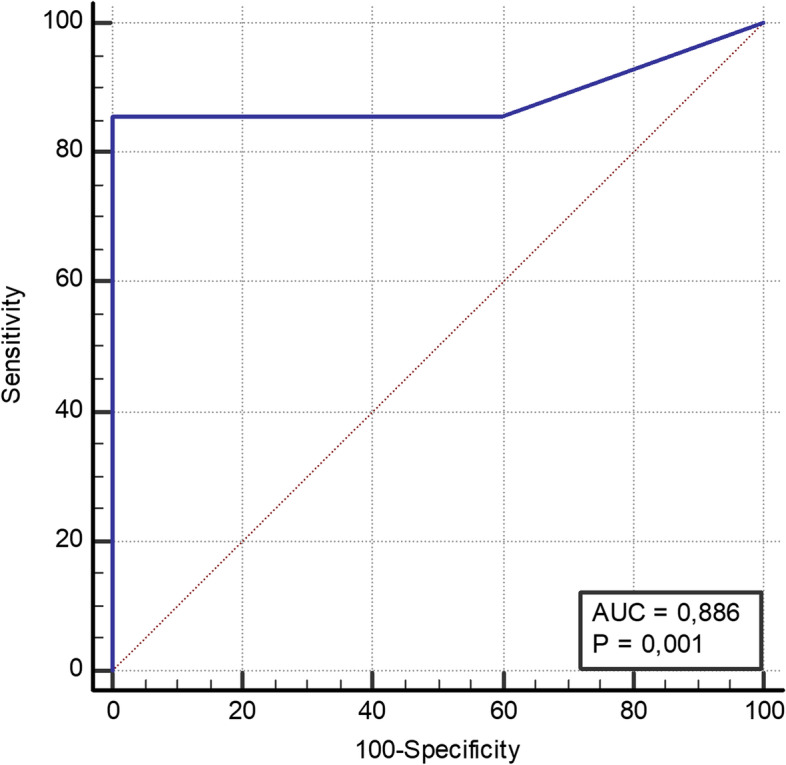
WBC. ROC-AUC analysis for WBC in relation to results of intraoperative microbiological culture during spacer implantation

**Fig. 4:**
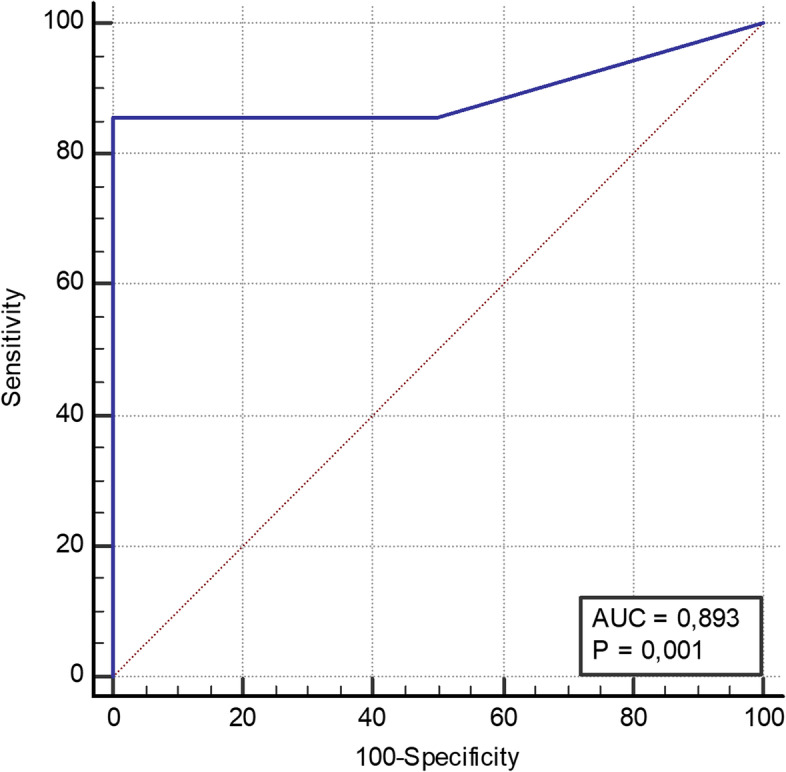
WBC. ROC-AUC analysis for WBC in patient with periprosthetic infection of the shoulder in relation to intraoperative microbiological culture during spacer implantation

Mean WBC was 27,800 leucocytes/mm^3^ (range 400-96,300). Results of WBC analysis are presented in Fig. 2. Compared to microbiologic culture from spacer implantation, the maximum Youden index (0.886) was a cut-off of 2600 leucocytes/mm^3^ with a sensitivity of 85.7% and a specificity of 100.0% (*p* < 0,001). ROC-AUC results are presented in Fig. 3. Mean WBC in patients with periprosthetic infections was 37,300 leucocytes/mm^3^ (range 400-96,300). The maximum Youden index (0,893) was a cut-off of 700 leucocytes/mm^3^ with a sensitivity of 85.7% and a specificity of 100.0% (*p* < 0,001). ROC-AUC results are presented in Fig. 4. Table 2 depicts detailed information on WBC.

### Interstage aspiration

**Fig. 5:**
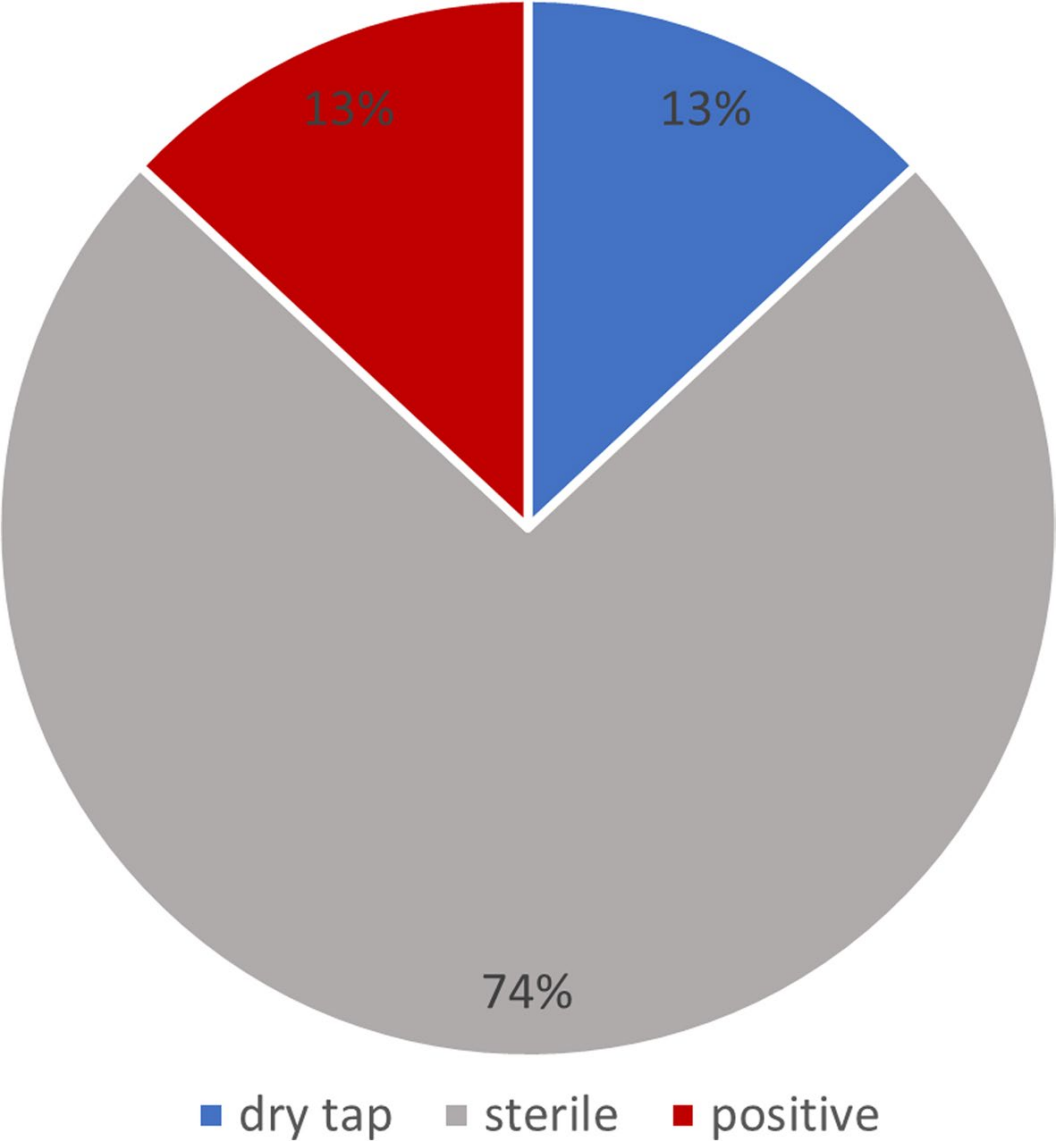
Microbiological cultures from IA. Results of microbiological cultures from IA (in % of all samples). For detailed information on detected bacteria see text

IA did not provide required minimum fluid volume in 3/33 cases. The remaining 30 cases showed positive cultures in 7%. Figure 5 depicts results of microbiological cultures from IA. Bacteria detected were Staphylococcus* epidermidis*, Paenibacillus *spp.* and Paracoccus *spp.* (one each). The sensitivity was 0.0% and the specificity was 88.5% with a PPV of 0.00 and a NPV of 0.92. Table 2 depicts detailed information on the microbiological results.

## Discussion

PA is a widely established method for the diagnosis of shoulder infection but its value is under debate. This debate is not at least caused by a sensitivity of PA reported within a wide range between 9 and 85% [9, 11, 12, 22]. The current study revealed a sensitivity of 58% for microbiological culture from PA. This is inferior to the reported sensitivity of intraoperative samples [16]. In particular, the detection rate of Cutibacterium *spp.* was low and CoNS were not detected at all. This is remarkable especially against the background that Cutibacterium *spp.* and CoNS are described as the main pathogens causing shoulder joint infections [12, 16, 22–24]. Their detection by aspiration is hampered as both are slow growing organisms and both are able to produce biofilms. Therefore, even while being present as sessile pathogens within a biofilm in a tissue, they might not be detectable in an aspirate in a planktonic state [25]. Second, Cutibacterium *spp.* are anaerobic bacteria and they are prone to be missed by culture due to inadequate preanalytic process such as suboptimal culture media or excessive long transport time [26]. Another controversially discussed aspect is the adequate period of incubation. Pottinger et al. described that only 86% of positive Cutibacterium *acnes* cultures were positive within 14 days [27]. In contrary, Frangiamore et al. considered true positive cultures to be positive within 4-6 days [28]. The incubation period of 14 days, used for the current study, is in accordance to other authors [8]. Notwithstanding this discussion, the high rate of false negatives (42%) of the current study stands in line with previous reports [7, 11, 22] and disqualifies microbiological cultures from PA to rule out infection. However, the herein high specificity of PA of 89% endorse PA as an important diagnostic pillar.

Alongside cultures, PA provides WBC. There is no consensus relating to a cut-off value for infection suspicion [29, 30]. To our best knowledge, very few studies exist that offer shoulder specific data. Nodzo et al. reported WBC in patients with periprosthetic C.* acnes* infections of the shoulder, hip and knee. Mean WBC for the PSI was 750 leucocytes/mm^3^. This was closest to values for the hip (500 leucocytes/mm^3^) [8]. Based on this study, 2018s International Consensus Meeting on periprosthetic joint infection in Philadelphia (ICM) suggested that PSI seems to be most likely comparable to low-grade periprosthetic hip infections and therefore recommended a cut-off of 3000 leucocytes/mm^3^ [31]. Strahm et al. who calculated WBC in 19 periprosthetic shoulder infections and suggested a threshold of 12,200 leucocytes/mm^3^ [32]. The mean WBC of 27,800 leukocytes/mm^3^ is clearly suspicious for infection. However, there was a wide range of WBC (400-96,300 leucocytes/ mm^3^). Based on our data, a cut-off value of 2600 leucocytes/mm^3^ revealed a sensitivity of 86% and specificity of 100% and therefore provides an excellent validation for PSI. For the subgroup of periprosthetic infections, a maximum Youden index was reached at a threshold of 700 leucocytes/mm^3^. While this result is based on a limited number of patients, it calls into question that periprosthetic infections of the shoulder come along with higher WBC than native or implant associated infections of the shoulder. Regardless of this point of discussion, our results implicate that WBC amongst various diagnostic parameters provided the best sensitivity to detect infection, yet a negative WBC cannot be used to rule out infection. We suggest that the direct adaption of values from hip infections to the shoulder might not be adequate. As our sample size was limited, further studies with enhanced sample size should follow to validate the current shoulder specific data on this important diagnostic parameter.

IA showed a devastating sensitivity of 0%. The problem of minimal detection rates is also known for periprosthetic infections of the hip and the knee. Boelch et al. described a sensitivity of 5% for the hip respectively 0% for the knee [18, 33]. In parallel, a shift from negative aspirations to low-virulent, biofilm-forming bacteria in intraoperative cultures was described [18]. It seems likely that this effect is even more relevant in shoulder infections as Cutibacterium *spp.* and CoNS are typical slow-growing, low-virulent organisms [34]. Our data supports these considerations insomuch as most false negative samples showed C. *acnes* in intraoperative cultures and the two cases with intraoperative detection of S.* epidermidis* had resulted in dry taps before. Our study revealed a good specificity of 89% for IA. Nevertheless, it is disputable whether diagnostic advantages are sufficient against the background of a sensitivity of 0%. Furthermore, an antibiotic suspension is recommended 2 weeks prior to aspiration in order to achieve best possible validity [35]. This however might increase the risk for infection persistence and the development of drug-resistant microbial strains [36]. Weighing up the diagnostic advantages and therapeutic disadvantages, we no longer perform IA in our clinic.

We acknowledge that this study has several limitations. The study design is retrospective. Including data from 2007, the definition of infection was not strictly based on 2018s ICM criteria. Due to the limited number of shoulder joint infections in general, the number of cases was limited. We included primary-, secondary- and periprosthetic infections. This heterogeneity empowers statistics but may cause a certain bias for PA, not for IA. Therefore subgroup analysis is supported as well.

## Conclusions

PA aspiration is an important pillar in the diagnosis of shoulder joint infection. Whilst sensitivity is moderate, specificity is high. WBC can be low even in cases with infection. Consequently, PA cannot absolutely rule out infection. However, we strongly recommend PA for its advantages of targeted antibiotic therapy in case of germ identification as well as its major impact on established infection scores. Surgeons should be aware that PA is likely to miss Cutibacterium *spp.* and CoNS. Empiric antibiotic therapy should therefore cover these bacteria even if aspiration showed negative microbiological cultures. In contrast, diagnostic value of IA seems negotiable and does not qualify for its routinely use.

## Data Availability

The datasets used and/or analyzed during the current study are available from the corresponding author on reasonable request.

## Abbreviations

AUC: Area under the curve
CoNS: Coagulase negative staphylococci
IA: Interstage aspiration
ICM: International Consensus Meeting on Periprosthetic Joint Infections
NPV: Negative predictive value
PA: Preoperative aspiration
PI: Primary infection
PMMA: Polymethylmetacrylate
PPV: Positive predictive value
PSI: Periprosthetic shoulder infection
ROC: Receiver operating curve
SI: Secondary infection
SJI: Shoulder joint infection
TSA: Total shoulder arthroplasty
WBC: Synovial fluid white blood cell count
